# Complete Uterine Torsion Secondary to a Massive Uterine Fibroid: A Case Report

**DOI:** 10.7759/cureus.90120

**Published:** 2025-08-14

**Authors:** Donna Salam, Mohamad Monif Assker, Diana Kokash, Olena Gorobchuk, Ahmed Al Kindi

**Affiliations:** 1 Radiology, Mohammed Bin Rashid University of Medicine and Health Sciences, Dubai, ARE; 2 Medical Imaging, Dubai Health, Dubai, ARE; 3 Radiology, Sheikh Khalifa Medical City, Abu Dhabi, ARE; 4 Radiology, Dubai Health, Dubai, ARE

**Keywords:** computed tomography, fibroid, magnetic resonance imaging, uterine leiomyoma, uterine torsion

## Abstract

Uterine torsion is an uncommon but life-threatening condition defined by the abnormal rotation of the uterus around its longitudinal axis, with the majority of cases occurring during the third trimester of pregnancy. While rare in nonpregnant patients, factors such as uterine fibroids, Müllerian anomalies, and pelvic adhesions can predispose individuals to this condition, leading to severe complications if not readily diagnosed and treated. We report the case of a 37-year-old nulliparous woman who presented with acute right-sided abdominal pain radiating to the back. CT and MRI evaluation revealed a massive subserosal uterine fibroid measuring 21.7 x 12.9 x 17.3 cm, causing complete uterine torsion with the left adnexa displaced to the right pelvis. Surgical intervention confirmed a 360-degree torsion, and the patient underwent successful detorsion and resection of the fibroid, which weighed 2.168 kg. Histopathological examination confirmed a benign leiomyoma with no evidence of malignancy. This case highlights the importance of early diagnosis and timely surgical intervention in managing uterine torsion secondary to fibroids. Diagnosing uterine torsion is challenging due to its nonspecific clinical presentation and overlap with other acute gynaecological emergencies. Imaging modalities such as ultrasound, CT, and MRI are valuable for preoperative diagnosis, but surgical exploration remains the gold standard for definitive diagnosis and management.

## Introduction

Uterine torsion is a rare but serious obstetric condition characterized by the abnormal twisting of the uterus, typically greater than 45 degrees around its long axis [1]. Dextrorotation accounts for approximately two-thirds of known cases [2]. Researchers have documented uterine torsion in all three trimesters, but about 85% of the cases occur during the third trimester [3]. Albeit exponentially rare, it can happen spontaneously in nonpregnant females [4]. Factors such as leiomyomas, Müllerian anomalies, fetal malpresentation, pelvic adhesions, and ligamentous laxity are all documented to increase the propensity of uterine torsion, eventually leading to significant complications such as shock, abruptio placentae, and maternal morbidity [5]. Researchers have consistently documented torsion rotation around 180 degrees, but in some cases, the rotation has reached up to 360 degrees [6]. Uterine fibroids, benign tumors that can distort normal uterine anatomy, are known risk factors that can increase the likelihood of torsion occurring, making this condition particularly notable in pregnant patients with fibroids. Clinicians often find diagnosis challenging due to symptom overlap with other acute obstetric emergencies, such as placental abruption, and they frequently make the diagnosis during surgical exploration rather than through imaging techniques. Prognosis for patients experiencing uterine torsion is highly variable, influenced by factors such as the degree of torsion, the gestational age at which it occurs, and the promptness of intervention. Researchers have documented maternal deaths, although they are rare in modern practice. We present the case of a uterine torsion secondary to a large fibroid along the following lines.

## Case presentation

A 37-year-old nulliparous woman presented to the emergency department complaining of right-sided lower abdominal pain radiating to the back. She had no history of fever, nausea, vomiting, weight loss, or abnormal bowel movements. The patient's menstrual cycles were regular, with no abnormal vaginal bleeding or discharge. During the physical exam, the patient was hemodynamically stable. Her abdomen was distended, with tenderness noted in the lower abdominal area. Her laboratory tests showed a slightly elevated C-reactive protein of 6.0 mg/l, while her complete blood count, liver function tests, and urine and stool analysis were all within normal limits. Her pregnancy test was also negative, and tumor markers (CA 125, CA 19-9, and alpha-fetoprotein (AFP)) were all within normal ranges (Table 1).

**Table 1:** Summary of laboratory results in the reported patient, with corresponding normal reference ranges.

Parameter	Result	Normal range
White blood cell count	7.8 × 10^3^/ *μ*L	3.6-11.0 × 10^3^/*μ*L
Hemoglobin	12.3 g/dL	13-17 g/dL
Platelets count	175 x 10^3^/ *μ*L	150-410 x 10^3^/ *μ*L
C-reactive protein (CRP)	6 mg/L	<5 mg/L
Serum creatinine	0.9 mg/dL	0.6-1.2 mg/dL
Urea	24 mg/dL	10-50 mg/dL
Prothrombin time	13.4 Secs	11 Secs
INR	1	0.8-1.1
APTT	33.0 Secs	28-41Secs
Aspartate aminotransferase (AST)	22 U/L	10-40 U/L
Alanine aminotransferase (ALT)	25 U/L	10-45 U/L
Alkaline phosphatase (ALP)	80 U/L	40-130 U/L
Total bilirubin	0.7 mg/dL	0.3-1.2 mg/dL
Albumin	4.2 g/dL	3.5-5.0 g/dL
Lipase	21 U/L	13-60 U/L
Beta-hCG	<5 mIU/mL	<5 mIU/mL

The radiology team performed imaging, and an abdominal ultrasound revealed a large mass measuring approximately 19 x 12 cm, with a heterogeneous echotexture, extending from the fundus and completely obscuring the uterus. The clinician could not properly visualise the ovaries.

**Figure 1:**
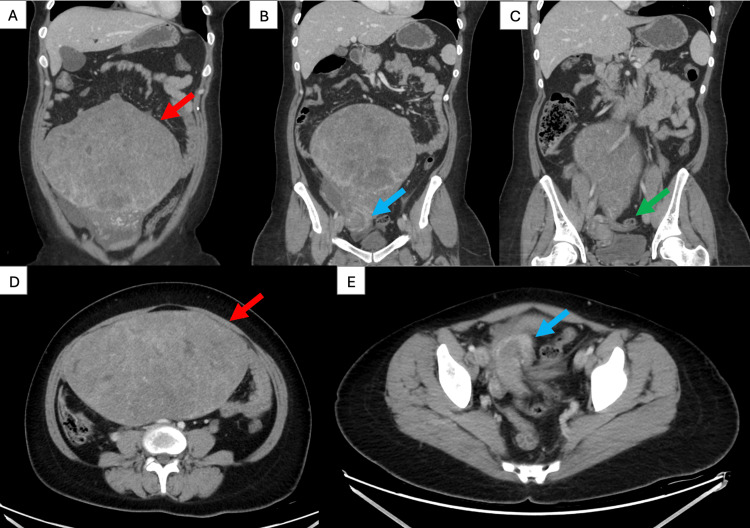
Computed tomography (CT) Contrast-enhanced cross-sectional imaging of the abdomen and pelvis showing a large superficial subserosal uterine mass, originating from the fundus and causing uterine torsion. The left adnexa is seen on the right side of the pelvis, with crossing of the left gonadal vessels through the torted uterine pedicle to the right side. (A, D) massive uterine fibroid, (B, E) uterine torsion caused by the fibroid, (C) left gonadal vessels crossing to the right side.

Contrast-enhanced cross-sectional imaging (CT) of the abdomen and pelvis showed a large superficial subserosal uterine mass originating from the uterine fundus, measuring approximately 21.7 x 12.9 x 17.3 cm. The soft tissue mass displayed smooth, regular contours and internal hypodensities. Post-contrast images showed a patchy enhancement pattern. Furthermore, this massive subserosal mass was causing uterine torsion. The surgeon observed the left adnexa situated on the right side of the pelvis, with the left gonadal vessels crossing through the tortuous uterine pedicle to the right side (Figure 1).

**Figure 2:**
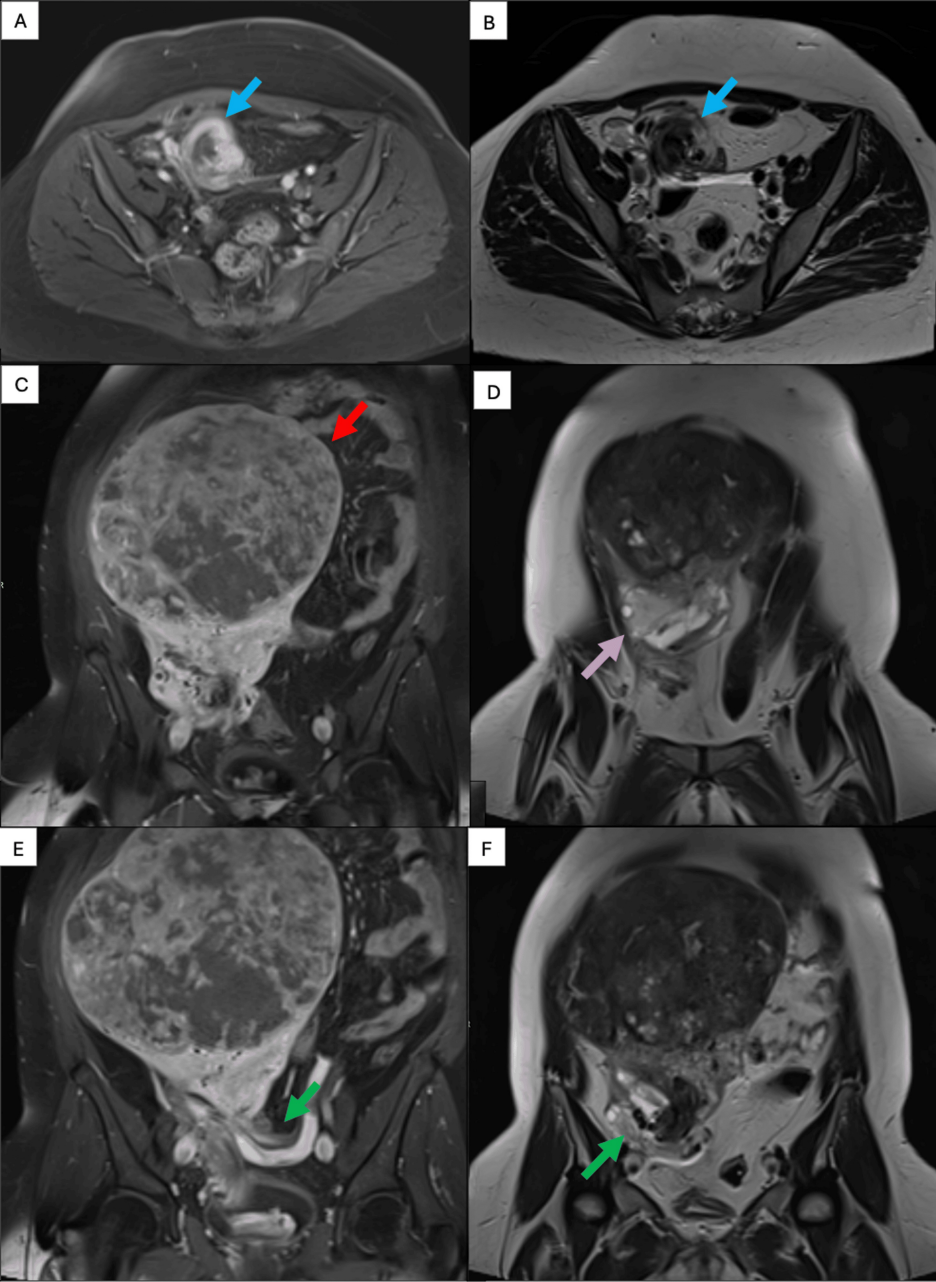
Magnetic resonance imaging (MRI) Magnetic resonance imaging (MRI) showing a large, well-defined fundal subserous soft tissue mass. The lesion is showing predominantly T2 hypointense signal with central scattered hyperintense signals and heterogeneous enhancement on post-contrast images. It is causing a mass effect on the bowels, displacing them superiorly and displacing the uterus inferiorly. The left adnexa is seen on the right side, with the crossing of the left gonadal vessels through the torted uterine pedicle to the right side. (A, B) uterine torsion caused by the fibroid, (C) massive uterine fibroid, (D) enlarged right ovary, (E, F) left gonadal vessels.

The radiology team performed magnetic resonance imaging (MRI) to further characterize the mass and exclude any malignancy features for appropriate surgical planning. They observed a large, well-defined fundal subserous soft tissue mass measuring 17 x 19 x 16 cm. The lesion showed predominantly T2 hypointense signal with central scattered hyperintense signals and heterogeneous enhancement on post-contrast images. It was causing a mass effect on the bowels, displacing them superiorly and the uterus inferiorly. The imaging team again demonstrated the left adnexa on the right side, and they observed the left gonadal vessels crossing through the tortuous uterine pedicle to the right side. These findings were suggestive of a uterine leiomyoma with features of cystic degeneration (FIGO classification - 6). The right ovary also appeared enlarged on MRI (Figure 2).

**Figure 3:**
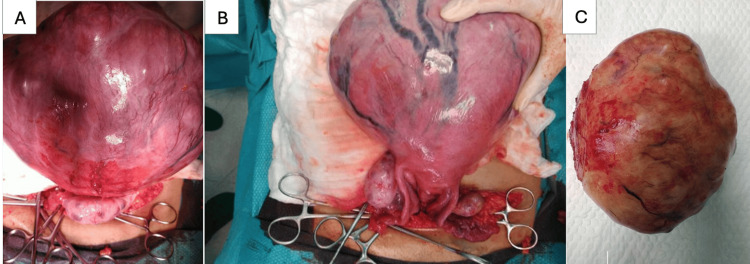
Gross images (A, B) The uterus, delivered outside the abdominal cavity, completely twisted three times with a large vascular-surfaced leiomyoma occupying the fundus. (C) Gross specimen of the excised uterine leiomyoma.

The patient underwent surgical resection of the massive lesion, measuring 22 x 12 cm and weighing 2.168 kg, seen grossly and caused complete uterine torsion. The uterus, which appeared to have twisted three times, was intraoperatively detorted and restored to its normal position (Figure 3). Histopathology examination of the mass revealed a uterine leiomyoma with no evidence of cellular atypia or mitotic activity. The postoperative course was unremarkable, and follow-up demonstrated full resolution with no adverse events.

## Discussion

The diagnosis of uterine torsion, particularly when secondary to uterine fibroids, poses significant challenges and is often established during laparotomy, as imaging modalities may not be readily available or utilized in time due to the urgency of the situation [7]. Patients typically present with acute abdominal pain, abdominal distension, and signs of shock, complicating the initial clinical assessment. Imaging techniques can assist in preoperative diagnosis, although their application is limited in many cases. Ultrasound, especially with Doppler interrogation, has proven effective for mapping and characterizing myomas and assessing organ vascularity; however, it is limited in its ability to definitively assess torsion. However, magnetic resonance imaging (MRI) is more sensitive than ultrasound for certain diagnoses, including uterine torsion [8]. The challenge often lies in timely access to these imaging modalities, especially in resource-constrained settings, leading to potential delays in diagnosis until surgical intervention is initiated. When uterine torsion occurs, it typically involves a twist of the uterus around its long axis, most commonly at the cervix-corpus junction, with twists ranging from 60° to over 720°. The diagnosis can be confirmed through a combination of physical examination and ultrasonography, which allows for the palpation of the round ligaments and identification of the irregularities of the uterine surface. Notably, when torsion leads to vascular obstruction, the subsequent symptoms may significantly exacerbate, underscoring the need for prompt diagnosis. Furthermore, a thorough differential diagnosis is critical, as conditions like uterine inversion or pelvic congestion syndrome may present similarly but require different management strategies.

The prognosis for patients experiencing uterine torsion is heavily influenced by the degree of torsion and the gestational age at which it occurs. Cases with torsion greater than 180 degrees and those occurring after 20 weeks of gestation are associated with higher risks of maternal morbidity and mortality [9]. While maternal deaths due to torsion are rare in modern medical practice, they have been documented, particularly in neglected or complicated cases. In the context of uterine fibroids, the presence of these tumors can complicate the clinical picture, potentially leading to delayed or misdiagnosis due to misleading symptoms such as abdominal pain or pressure. This diagnostic challenge may result in a worse prognosis if not managed promptly. The potential for serious sequelae exists, with literature indicating a history of maternal losses associated with torsion. The recurrence of torsion in subsequent pregnancies is noted, although the incidence is considered low [10].

Uterine torsion, particularly when associated with uterine fibroids, can lead to a variety of serious complications. One of the most common complications observed is postembolization syndrome, which is characterized by symptoms such as mild fever, pain, and vaginal expulsion of fibroids following treatment [11]. During surgical interventions for uterine torsion, complications can arise, including irreversible ischemia of the uterus and adjacent pelvic organs, which may necessitate hysterectomy or oophorectomy if necrosis occurs [7]. Post-embolization symptoms typically resolve within a few days but may include pelvic pain, cramping, nausea, and fatigue, highlighting the need for monitoring and supportive care post-treatment [12]. 

## Conclusions

Our case highlights a rare and unique case of uterine torsion secondary to a massive fibroid. This life-threatening condition, although uncommon, should be considered by physicians. While surgical exploration remains the gold standard for diagnosis, various imaging techniques can assist in accurate evaluation and expedite the diagnostic process.
